# Comparative analysis of blood whole transcriptome profiles in Yili horses pre- and post-5000-meter racing

**DOI:** 10.3389/fgene.2025.1651628

**Published:** 2025-08-29

**Authors:** Yi Su, Wanlu Ren, Shikun Ma, Jun Meng, Xinkui Yao, Yaqi Zeng, Zexu Li, Luling Li, Ran Wang, Jianwen Wang

**Affiliations:** ^1^ College of Animal Science, Xinjiang Agricultural University, Xinjiang, China; ^2^ Xinjiang Key Laboratory of Equine Breeding and Exercise Physiology, Urumqi, China

**Keywords:** Yili horses, exercise stress, whole transcriptome sequencing, differentially expressed genes, signaling pathways

## Abstract

This study employed Yili horses participating in a 5000-meter race as a model to investigate exercise-induced gene expression alterations in peripheral blood using whole transcriptome sequencing. Jugular vein blood samples from the three leading horses were collected pre- and immediately post-race, yielding 2,171 differentially expressed mRNAs (2,080 upregulated, 91 downregulated), 4,375 differentially expressed LncRNAs (4,354 upregulated), and 68 differentially expressed circRNAs (64 upregulated). GO/KEGG analyses demonstrated significant enrichment of differential mRNAs in transmembrane transport function and pivotal signaling pathways (cAMP, MAPK, PI3K-Akt). Differential lncRNAs targeted neuro-signaling pathways (e.g., Neuroactive ligand-receptor interaction, Calcium signaling) and developmental regulators (stem cell pluripotency). Source genes of circRNAs were enriched in axon guidance and immune-related T cell receptor signaling. Molecular functions converged on transporter/receptor activity (mRNA/lncRNA) and nucleic acid/GTP binding (circRNA source genes). The protein-protein interaction analysis identified ten central genes within the heat shock protein family, such as *HSP90AA1* and *HSPA4*. Notably, significant upregulation of *HCN4, IGF1, PTHR1,* and *FGF23* indicated their potential roles in modulating cardiac rhythm, promoting tissue repair, and maintaining calcium-phosphorus homeostasis during exercise adaptation. This study provides comprehensive overview of transcriptomic regulatory mechanisms in the blood of Yili horses, offering a molecular framework for advancing understanding of physiological adaptation to exercise and optimizing equine exercise protocols.

## 1 Introduction

As a locally developed breed in China, the Ili horse demonstrates outstanding genetic traits and athletic performance within China’s horse racing industry. However, a significant performance gap remains between this breed and internationally renowned speed horse breeds. Therefore, this study focuses on the Ili horse as the research subject, which is instrumental in accelerating the development process of domestically-bred speed horses within China. The sport horse field is a critical sector within the modern equine industry, and their competitive performance directly impacts economic outcomes. Accurate physiological monitoring is essential to maintaining the competitive health and welfare of sport horses. In recent years, substantial datasets on hematological, physiological, and biochemical parameters across various breeds have been collected (Harari et al., 2024; Giers et al., 2024; Masko et al., 2021), elucidating the dynamic metabolic, immune, and muscular responses to exercise-induced stress (Huangsaksri et al., 2024). Despite this progress, a systematic investigation into exercise-induced global gene expression alterations and the underlying regulatory networks in horses remains lacking.

Exercise, as a potent physiological stimulus, remodels systemic metabolism and adaptive responses through the activation of multifaceted molecular regulatory networks. Evidence indicates that transcriptional regulation triggered by exercise includes essential biological pathways, including energy metabolism, oxidative stress, and inflammatory signaling (Yang et al., 2013; Reitzner et al., 2024). For instance, gene expression analyses in the skeletal muscle of thoroughbreds post-intense exercise revealed marked alterations in genes associated with transcription factor activity, including oxidoreductases and protein-binding elements (Farries et al., 2020). Similarly, transcriptomic profiling of Arabian horses participating in endurance races indicated dynamic shifts in gene networks governing cell migration and tissue repair (Cappelli et al., 2018). Investigations into equine exercise physiology further demonstrate that acute exercise rapidly upregulates genes within the glycolytic pathway, TCA cycle, and oxidative phosphorylation, emphasizing the transcriptional basis of adaptive capacity (Bryan et al., 2017). While epigenetic research has identified correlations between DNA methylation patterns in sport horse blood and key signaling pathways such as PI3K-Akt (Stefaniuk-Szmuki et al., 2018), comprehensive transcriptomic analyses remain limited. Whole-transcriptome approaches enable simultaneous characterization of mRNA expression dynamics alongside the intricate regulation exerted by non-coding RNAs and alternative splicing events, offering a broader framework to elucidate the molecular basis of exercise adaptation.

In this study, peripheral blood samples were collected from Yili horses participating in a 5000-meter race, both pre- and immediately post-race, and subjected to high-throughput whole transcriptome sequencing to comprehensively profile exercise-induced gene expression alterations. Differentially expressed genes (DEGs) and associated regulatory networks were identified, enabling the enrichment of signaling pathways implicated in energy metabolism, inflammatory processes, and tissue repair. These findings contribute to a molecular-level understanding of physiological adaptation in sport horses and support the development of more effective training regimens and health management protocols.

## 2 Materials and methods

### 2.1 Sample collection

For the 5000-meter speed race of Yili horses conducted in Zhaosu County, Xinjiang’s Ili Kazakh Autonomous Prefecture, all participating horses (24 in total) underwent veterinary health examinations (lameness screening) and breed verification (via passport inspection) prior to the competition. All entrants were required to arrive at the competition terrain by 18:00 on the day preceding the event. At 20:00 that same day, 5 mL jugular venous blood samples were collected from all horses using EDTA anticoagulant vacuum tubes. Within 5 min post-race, venous blood samples were again drawn from the jugular veins of the top three finishers (Group A). For this study, pre-race blood samples corresponding to these top three performers were selected as experimental specimens (Group B). Detailed information on the horses is presented in Table 1. All collected samples were immediately cryopreserved in liquid nitrogen for subsequent analysis.

**TABLE 1:** Equine details.

​Horse ID​	​Age (Y.O.)	Sex	Height at withers (cm)	Trunk length (cm)	Breed	Race results
1	4	Stallion	153	150	Yili Horse	5′23″704
2	4	Stallion	152	147	Yili Horse	5′32″530
3	4	Stallion	153	162	Yili Horse	5′41″339

### 2.2 Library preparation

Total RNAs were extracted from blood samples, and mRNAs were enriched using mRNA capture magnetic beads. The enriched mRNAs were subsequently fragmented and used for cDNA synthesis. Following cDNA purification with Hieff NGS^®^ DNA Selection Beads, target fragments were screened, and the size-selected fragments were subjected to PCR amplification to construct libraries with an insert size of 350–400 bp High-quality libraries were submitted to Novogen (Beijing, China) for 150 bp paired-end sequencing on the Illumina NovaSeq 6,000 platform (Illumina, CA, USA).

### 2.3 Bioinformatics analysis

FastQC (fastqc_v0.11.8) was employed to assess the quality metrics of raw Illumina sequencing data. Adapter sequences and low-quality reads were removed using Fastp (fastp 0.23.1), yielding high-quality clean reads. Bismark (version 0.24.0) was subsequently applied to map the clean reads to the *Equus Caballus* reference genome (EquCab3.0). Reads successfully aligned to the reference genome were designated as target sequences for subsequent standardized and customized analyses.

Filtering parameters included the elimination of reads containing adapter contamination, those with terminal base quality scores below 3, or ambiguous bases (N). A sliding window approach, with a 4-base window and a quality threshold of 15, was used to truncate reads at the point where the average quality within the window fell below the threshold. Reads shorter than 36 nt post-trimming or lacking valid pairing were excluded from further analysis (Ana et al., 2016).

### 2.4 GO and KEGG enrichment analyses

GO and KEGG enrichment analyses of DEGs and differentially expressed LncRNA target genes were conducted using ClusterProfiler (v3.10.1) and KOBAS (v2.0). GO analysis included three functional categories: biological process (BP), cellular component (CC), and molecular function (MF). Significantly enriched GO terms and KEGG pathways were identified based on a threshold of p adj <0.05.

### 2.5 Protein-protein interaction (PPI) network analysis

Based on the intersection of DEGs and known protein interaction pairs from the STRING database, a PPI network was assembled. Homologous protein interaction relationships were integrated into the network, which was subsequently visualized with Cytoscape (v3.8.0). The cytoHubba plug-in was applied to extract the top 10 core genes within the network.

### 2.6 RT-qPCR

To validate the transcriptome sequencing results, seven mRNAs were randomly selected for real-time quantitative PCR (qPCR) analysis (Hutchinson, 2015). Extract total RNA from whole-blood samples using the TRIZOL reagent (Thermo Fisher Scientific, Catalog # 15596026, USA). Mix 300 μL of whole blood with 900 μL of TRIZOL thoroughly and let it stand for 5 min. Then, add 200 μL of chloroform (Sinopharm Chemical Reagent Co., Ltd., Catalog # 10006818, China), centrifuge at 12,000 rpm at 4 °C for 10 min. Collect the supernatant, add 500 μL of isopropyl alcohol (Sinopharm Chemical Reagent Co., Ltd., Catalog # 80109218, China) to precipitate the RNA, wash it with 75% ethanol, and dissolve it in nuclease-free water (Servicebio, Catalog #G4700, China). Measure the RNA concentration and purity using the NanoDrop 2000 spectrophotometer (Thermo Fisher Scientific, Wilmington, DE, USA) with an A260/A280 ratio of 1.8–2.0. Then, 2 μg of total RNA was reversely transcribed into cDNA using HiScript^®^ Q RT SuperMix kit (TOYOBO Life Science, Cat# FSQ-101, Japan) in a 20 μL reaction mixture under the condition of 37 °C for 15 min and 98 °C for 5 min in a Veriti thermal cycler (Thermo Fisher Scientific, Waltham, MA, USA). The amplification was carried out using ChamQ SYBR qPCR Master Mix (TOYOBO Life Science, Cat# QPK-201, Japan) on a TL-988 real-time PCR system (Xi’an Tianlong Technology Co., Ltd., China). Primers with specific specificity were designed using Primer 5 software (Table 2), and the 20 μL reaction mixture consisted of 10 μL of pre-mix solution, 0.4 μM forward and reverse primers, and 1 μL of cDNA template. The amplification procedure involved two stages: Stage 1: 95 °C for 30 s for primer denaturation; Stage 2 (40 cycles): 95 °C for 15 s for denaturation, followed by 60 °C for 30 s for annealing/extension. Stage 3 (Melting Curve): From 65 °C to 95 °C, collect one fluorescence signal per 0.5 °C increment. All samples have three technical repeats. Gene relative expression levels were calculated using the 2^−ΔΔCT^ method, with GAPDH serving as the reference gene for normalization (Supplementary Table S1).

**TABLE 2:** RT-qPCR primers.

Gene name	Primer sequences (3'→5')	Product size (bp)
*GAPDH*	F: CAT​CAA​ATG​GGG​CGA​TGC​TG	158
	R: GGT​TCA​CGC​CCA​TCA​CAA​AC	
*CACYBP*	F:AGTCCCCACTGAGAATGTGC	127
	R: GCC​TTC​CAC​AGA​GAT​GGG​TT	
*DNAJB1*	F: CGT​CGG​ACG​AGG​AGA​TCA​AG	160
	R: CCG​AAG​CGG​TCG​AAA​ATG​TC	
*HSP90AA1*	F: CAC​AGG​TGA​GAC​CAA​GGA​CC	105
	R: CAG​TAC​TCG​TCG​ATC​GGC​TC	
*LOC100054696*	F: CGA​TGT​TTT​GGG​GGT​CAA​ACC	81
	R: GGG​TGG​TAC​TTC​AAG​GCC​AG	
*LOC100072672*	F: CAA​CCC​GGA​GGA​CAA​GTA​CC	103
	R: ACC​GCA​AGG​CAT​TTC​ATC​T	
*PTGES3*	F: ACA​TGG​GTG​GTG​ATG​AGG​ATG	117
	R: CCG​GTG​ATG​GTA​ACA​TTC​CTT	
*SLC5A3*	F: GGA​AGC​GCT​GCA​ATG​AAC​AA	136
	R: TTG​ATG​AAG​CCT​GGC​CTG​TT	

## 3 Results

### 3.1 Sequencing quality analysis

Blood transcriptome data were obtained from six samples, comprising early-stage (Group B) and late-stage (Group A) time points of the race. Quality control metrics, summarized in Table 3, indicated that Group B and Group A yielded 238,441,444 and 246,501,422 clean reads, respectively. Q20 and Q30 values exceeded 98.00% and 94.00%, while GC content ranged from 46.35% to 49.92%,Mapped reads ≥90% for all samples. Both groups demonstrated sequencing pass rates above 90%, confirming the robustness and reliability of the datasets for further analyses.

**TABLE 3:** Sequencing quality and read counts.

Sample	Raw reads	Clean reads	Error rate	Q20 (%)	Q30 (%)	GC content/(%)	Mapped reads
B1	78,769,258	76,747,064	0.01	98.54	96.12	49.92	71,729,558 (93.46%)
B2	86,281,582	84,453,076	0.01	98.1	94.88	49.61	76,454,194 (90.53%)
B3	78,833,206	77,241,304	0.01	98.67	96.42	49.35	72,705,768 (94.13%)
A1	83,030,990	81,319,316	0.01	98.61	96.32	47.76	75,915,707 (93.36%)
A2	85,764,908	82,977,310	0.01	98.43	95.95	46.69	74,818,193 (90.17%)
A3	84,442,934	82,204,796	0.01	98.66	96.35	49.41	76,141,631 (92.62%)

### 3.2 Gene expression profiling

**FIGURE 1:**
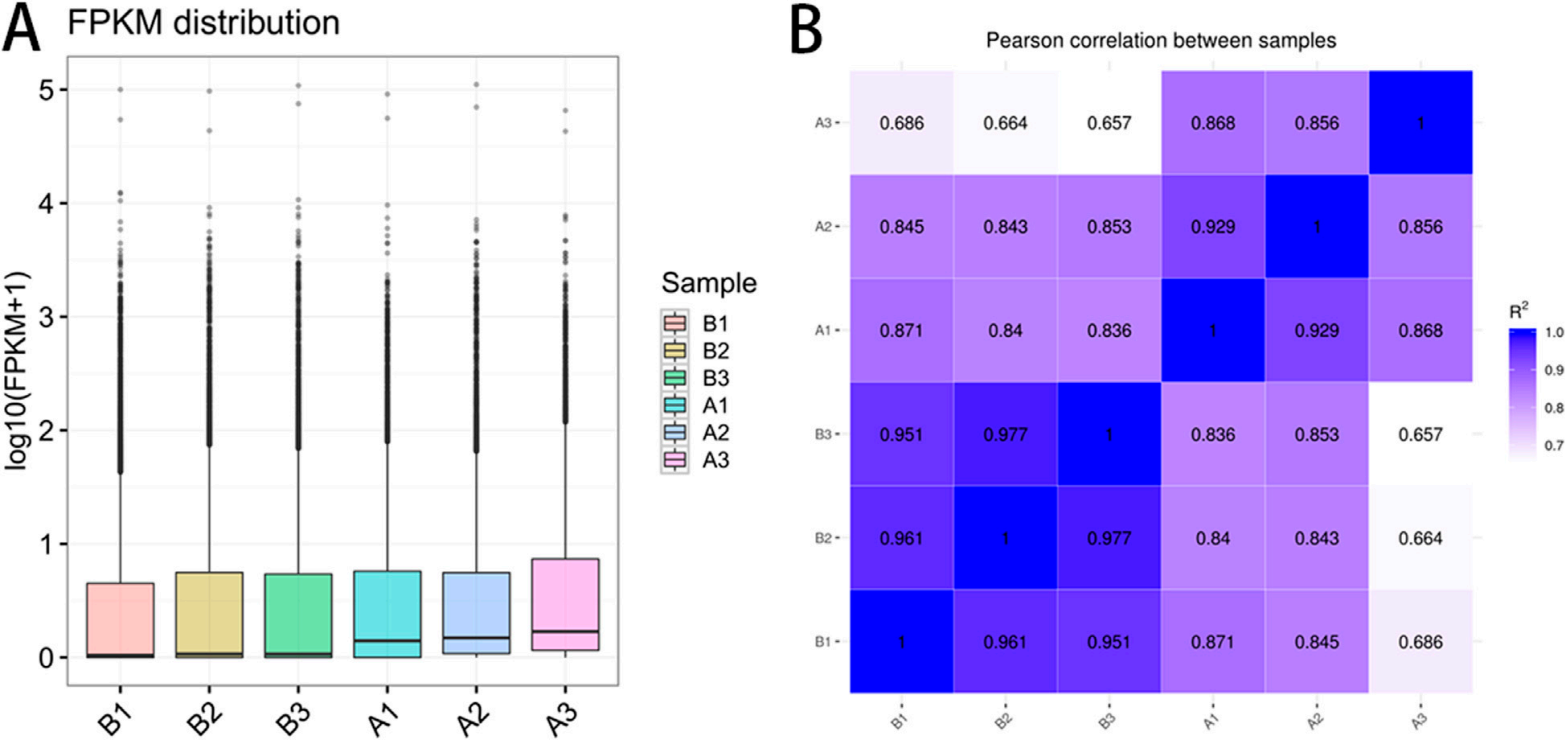
mRNA expression level pre- and post-race. **(A)** mRNA expression level; **(B)** Pearson correlation coefficient diagram. Note: **(A)** illustrate the distribution of gene expression levels across six biological replicates (B1-B3, A1-A3), with box plots showing the transformed expression values after logarithmic transformation by base 10 (FPKM +1). The vertical axis represents the transformed expression values, while the boxes represent interquartile range (IQR). The lines extend beyond 1.5 times IQR for outliers. **(B)** Display a heatmap depicting sample-wise correlations in expression, with color depth indicating the strength of Pearson correlation coefficient (R^2^) (ranging from light purple 0.7 to dark blue 1.0). Diagonal lines indicate self-correlation within the same samples (R^2^ = 1.0).

The FPKM distribution, visualized via box plots (Figure 1A), reflected consistent global gene expression across samples. The x-axis denoted sample identifiers, while the y-axis represented log_10_(FPKM). Minimal dispersion and comparable expression ranges among samples supported the data’s reproducibility. Pearson correlation analysis (Figure 1B) further indicated clear intergroup distinction, with low intra-group variability, validating the appropriateness and reliability of sample selection for comparative transcriptomic analysis.

### 3.3 Analysis of differential mRNAs, LncRNAs, and circRNAs

**FIGURE 2:**
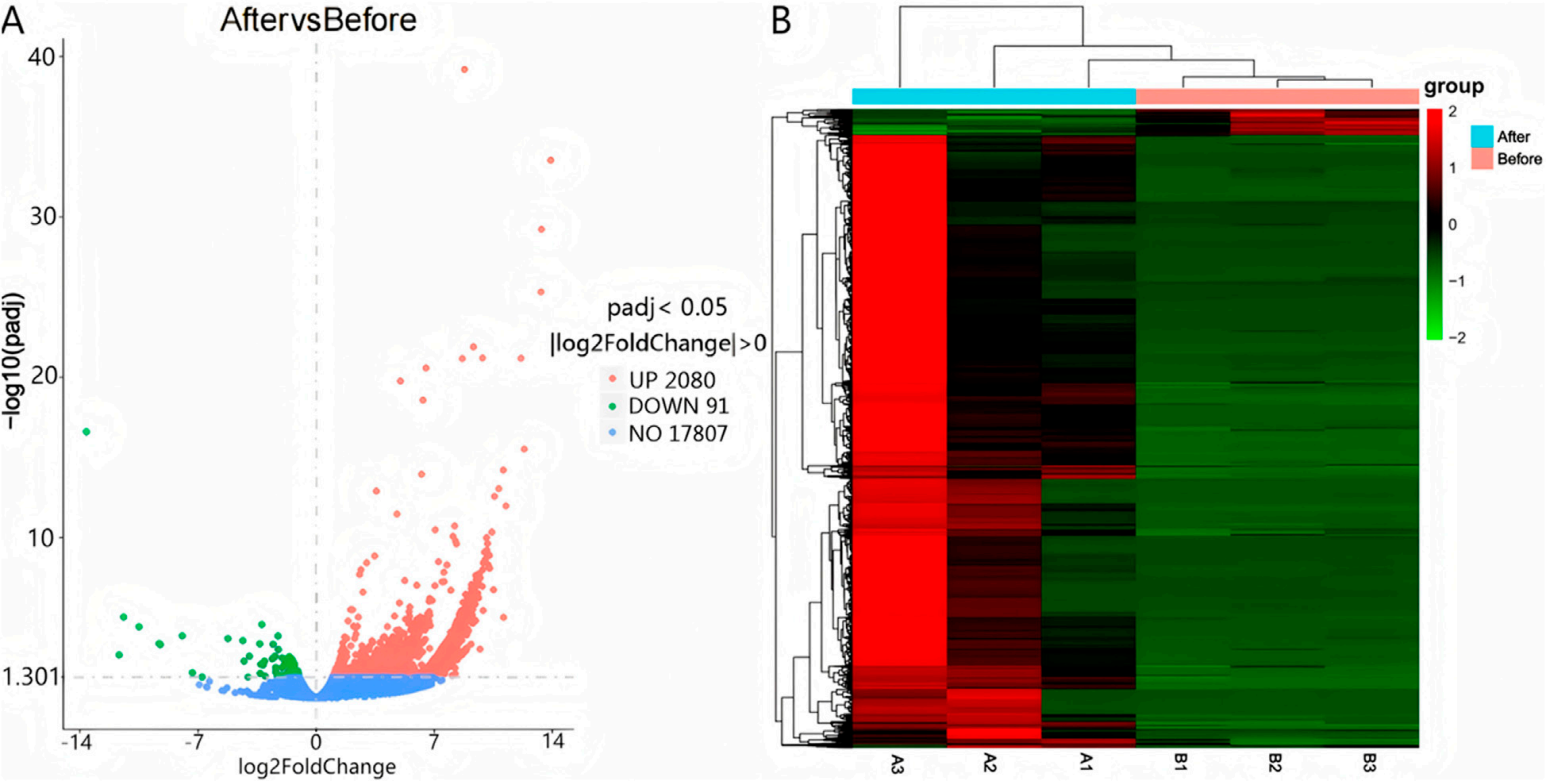
Volcano plot **(A)** and heatmap **(B)** illustrating mRNA expression differences between group B and group A.

**FIGURE 3:**
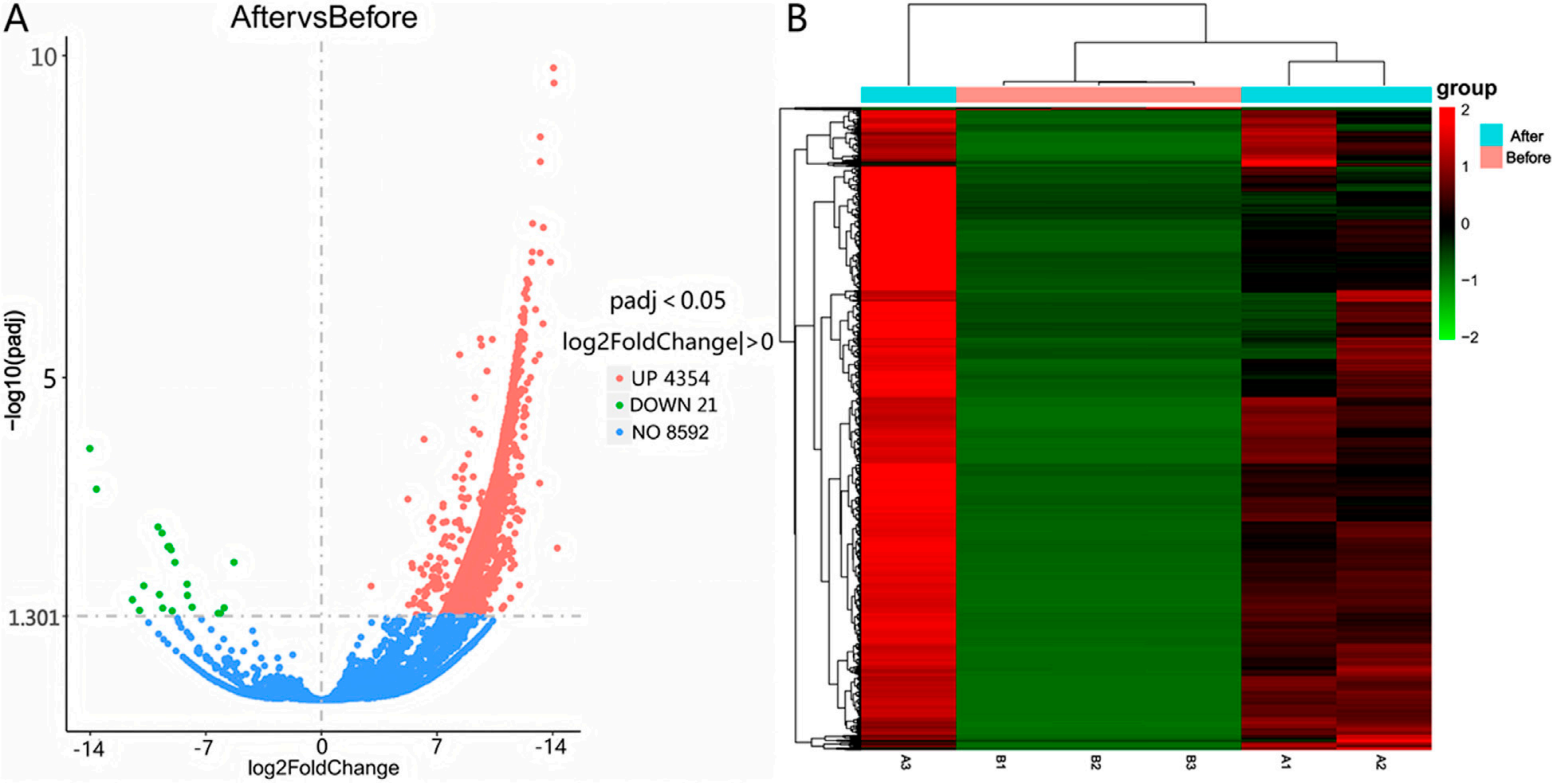
Volcano plot **(A)** and heatmap **(B)** illustrating LncRNA expression differences between group B and group A.

**FIGURE 4:**
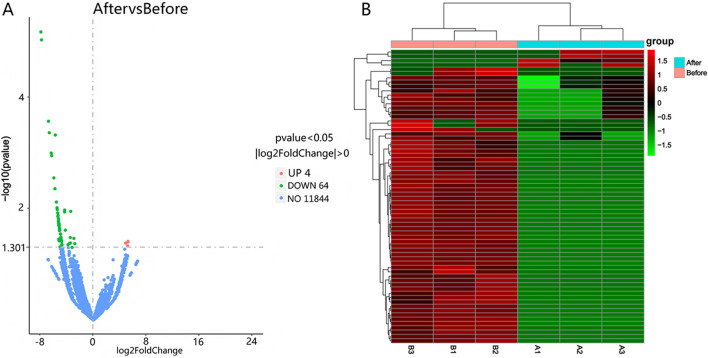
Volcano plot **(A)** and heatmap **(B)** illustrating circRNA expression differences between group B and group A.

Differential expression was determined using |log2(Fold Change)| > 0 and Padj <0.05 as selection thresholds. Comparative analysis between Group A and Group B identified 2,171 differentially expressed mRNAs, including HCN4, IGF1, PTHR1, and FGF23, with 2,080 upregulated and 91 downregulated (Figure 2A). A total of 4,375 differentially expressed LncRNAs were detected, comprising 4,354 upregulated and 21 downregulated transcripts (Figure 3A). Additionally, 68 circRNAs displayed differential expression, with 4 upregulated and 64 downregulated (Figure 4A). The cluster analysis results demonstrated that the differential mRNAs, LncRNAs, and circRNAs in blood samples before and after the competition, as shown in Figures 2B, 3B, 4B, were highly reproducible, revealing significant differences between the groups.

### 3.4 GO and KEGG analyses of differential mRNAs, LncRNAs, and circRNAs

**FIGURE 5:**
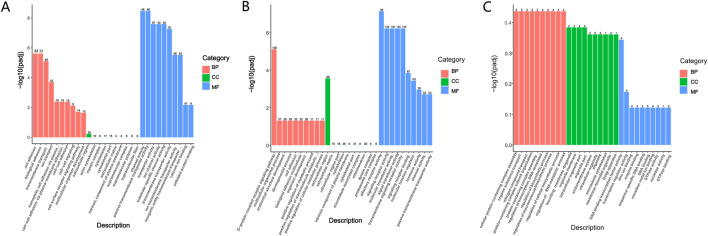
GO enrichment analysis. **(A)** Differential mRNAs; **(B)** differential LncRNAs; **(C)** differential circRNAs.

**FIGURE 6:**
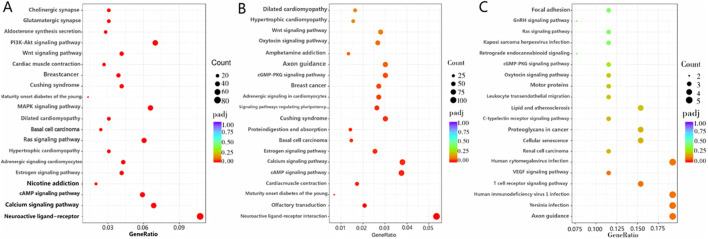
KEGG enrichment analysis. **(A)** Differential mRNAs; **(B)** differential LncRNAs; **(C)** differential circRNAs.

GO enrichment analysis and database annotation (Figure 5A) identified significant (P < 0.05) enrichment of differential mRNAs in 17 BP and 26 MF categories. Among the GO terms, transmembrane transport, transporter activity, and transmembrane transporter activity were prominently represented. KEGG pathway analysis (Figure 6A) further revealed that DEGs were enriched in signaling cascades including cAMP, MAPK, and PI3K-Akt pathways.

GO enrichment analysis was subsequently performed on differential LncRNAs (Figure 5B), revealing significant enrichment (P < 0.05) across 11 BP, 1 CC, and 27 MF categories. The enriched BPs were the G-protein-coupled receptor signaling pathway, multicellular organismal process, and cell adhesion. The sole enriched CC was the extracellular region. Within MF, enriched terms included transporter activity, molecular function regulator, signal transducer activity, molecular transducer activity, and signaling receptor activity. KEGG pathway analysis (Figure 6B) further demonstrated significant enrichment of differential LncRNAs in pathways including Neuroactive ligand-receptor interaction, cAMP signaling, Calcium signaling, regulation of stem cell pluripotency, and cGMP-PKG signaling.

GO-based functional annotation of the differentially expressed circRNAs (Figure 5C) identified enrichment across 68 corresponding source genes. In the BF category, annotations were primarily associated with nucleic acid metabolic processes and regulation of biosynthetic activity. CC terms predominantly involved cellular and organelle-related localizations. Within the MF category, functional associations were concentrated in GTP binding, DNA binding, and related molecular interactions. KEGG pathway enrichment analysis further revealed significant representation in signaling pathways such as Axon guidance and T cell receptor signaling (Figure 6C). GO and KEGG enrichment can be found in the supplementary material (Supplementary Table S3; Supplementary Table S4).

### 3.5 PPI network analysis

**FIGURE 7:**
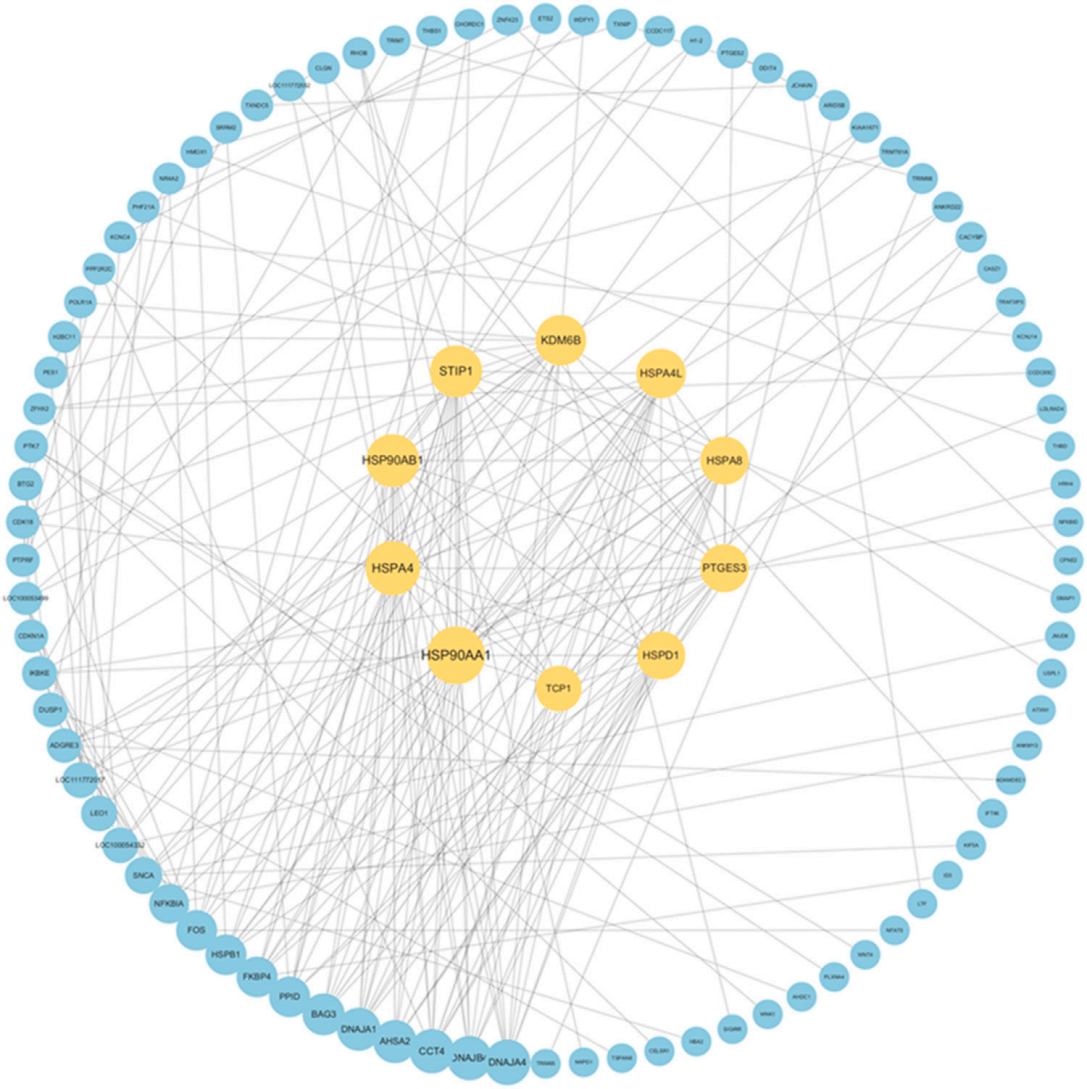
PPI network of DEGs and identification of core genes. Note:The outer blue nodes indicate differentially expressed genes, while the inner yellow nodes (n = 10) denote hub genes, with lines representing significant co-expression relationships (Pearson |r| > 0.9).

A PPI network was established based on DEGs identified in the blood of Yili horses pre- and post-race (Figure 7), enabling the identification of the top 10 core genes: *HSP90AA1, HSPA4, HSP90AB1, STIP1, KDM6B, HSPA4L, HSPA8, PTGES3, HSPD1,* and *TCP1.* Each exhibited markedly elevated expression.

### 3.6 RT-qPCR verification

**FIGURE 8:**
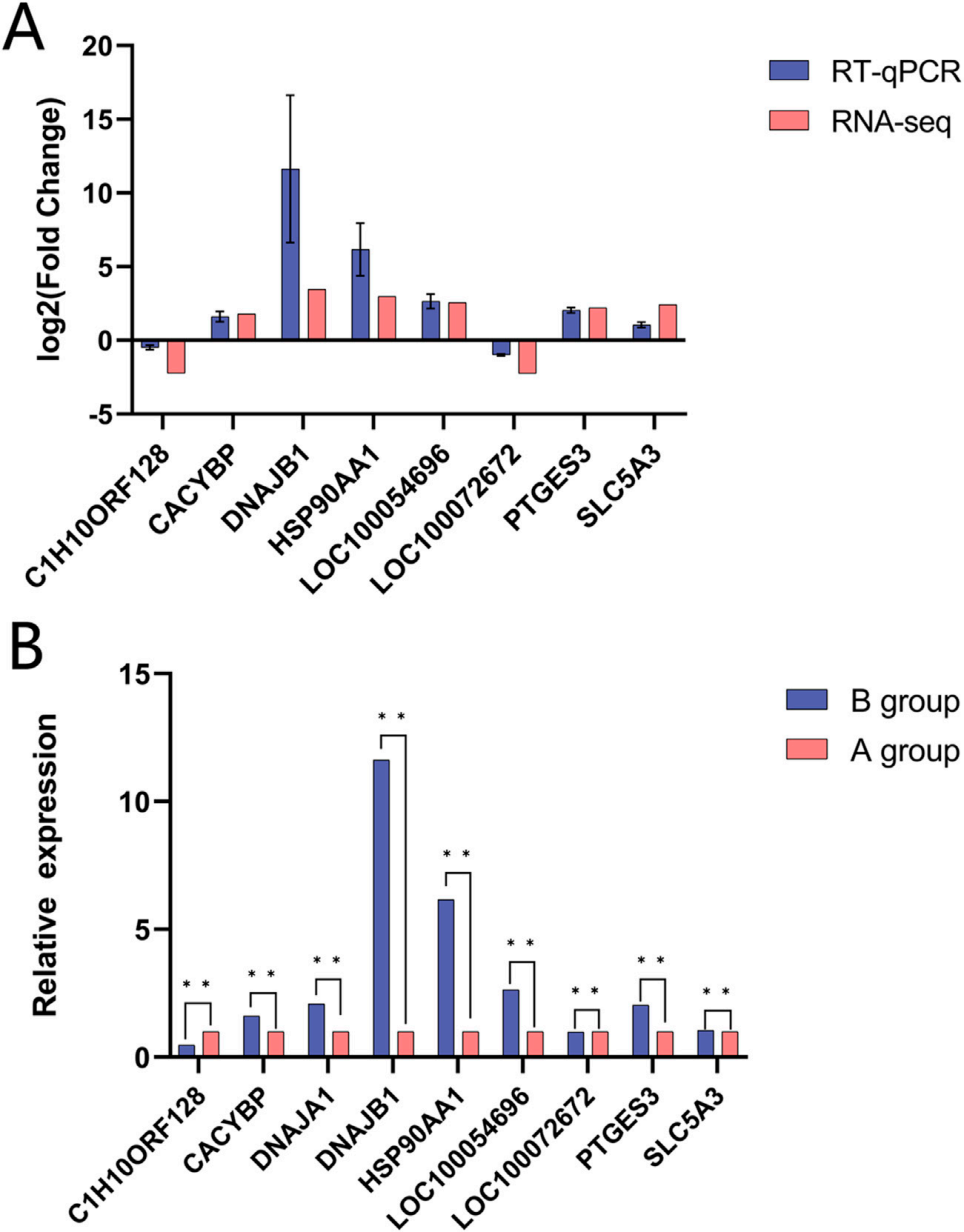
Validation of RNA-seq data using RT-qPcR for Yili horses, Data are expressed as mean t standard error of mean (SEM). **(A)** Indicate Log2Fcobtained by RNA-seq and RT-qPCR of DEGs. **(B)** Represent the relative expressions of DEGs, in RT-qPcR.

The fluorescence quantification outcomes of the seven mRNAs, as depicted in Figure 8, exhibited concordance with the RNA-seq results, confirming the reliability of the transcriptomic data analysis. This consistency validated the RNA-seq dataset as a sound basis for subsequent investigations (Supplementary Table S1).

## 4 Discussion

During racing, equine physiological adaptation requires coordinated modulation of multiple signaling pathways to maintain systemic homeostasis. In this study, whole transcriptome sequencing of pre- and post-race blood samples from Yili horses identified 2,171 differentially expressed mRNAs. These transcripts represent potential targets for regulating exercise-related responses in horses. Notably, *HCN4, IGF1, PTHR1,* and *FGF23* emerged as shared differential mRNAs, with all four exhibiting significant post-race upregulation relative to the remaining 2,077 transcripts. This expression pattern suggests a potential link between elevated mRNAs expression and enhanced exercise capacity. As a “central pacemaker” within the sinoatrial node (SAN), *HCN4* responds to increased cAMP induced by physical activity via its C-terminal cyclic nucleotide binding domain (CNBD) (Porro et al., 2020), enhancing the pacemaker current (If) to elevate heart rate and maintain cardiac output stability during 5000-meter races. This mechanism is further substantiated by the accumulation of cAMP signal pathways and their direct relevance to the protection against exercise-induced arrhythmias (Lin et al., 2024). *HCN4* plays a central role in regulating cardiac rhythmicity. Studies in rabbit models have demonstrated that increased *HCN4* expression supports sinoatrial node recovery and mitigates arrhythmogenic events, highlighting its contribution to heart rate modulation and myocardial energy balance (Tuerxun et al., 2024). Yukihiro et al. (Saito et al., 2022) demonstrated that targeted integration of the *HCN4* gene into the AAVS1 locus via TALEN-mediated editing induced *HCN4* channel protein expression and elevated IF current, confirming the role of *HCN4* overexpression in enhancing the pacing capacity of cardiomyocytes. Post-race analysis revealed a marked upregulation of *HCN4* expression, suggesting that exercise may trigger activation of the *HCN4* gene, thereby increasing channel density on the cardiomyocyte membrane and modulating cardiac pacing to support enhanced physical performance. Nevertheless, an excessive elevation in heart rate may compromise cardiac output or induce arrhythmogenic events, ultimately impairing exercise capacity and heightening cardiovascular risk. *IGF1* contributes to the synthesis of proteoglycans, supports cellular viability, and attenuates cytokine-driven catabolic processes (Li et al., 2024; Jin et al., 2025; Menetrey et al., 2000). IGF1 elevation facilitates dystrophin synthesis by activating the PI3K-Akt signaling pathway, thereby reducing muscular membrane damage (Song et al., 2021). The corresponding protein plays a regulatory role in maintaining cellular metabolic homeostasis and promoting tissue repair mechanisms (Christopoulos et al., 2015). In this study, post-race upregulation of the DEG *IGF1* in Yili horses suggests a potential involvement in tissue repair. Vigorous exercise during racing is known to induce muscle cell damage (Flick et al., 2021; Wilson et al., 2023); in response, elevated *IGF1* expression may contribute to cellular repair and preservation of cytoskeletal integrity (Mir et al., 2017). Activation of the cAMP signaling pathway is triggered when *PTHR1* binds to *PTH* or *PTHrP* (Singh et al., 2005; Gesty-Palmer et al., 2006), promoting renal calcium reabsorption while concurrently suppressing phosphate reabsorption (Liu et al., 2012). This cascade also enhances bone resorption and intestinal calcium uptake, contributing to the maintenance of systemic calcium levels (Fu et al., 2021). During high-intensity exercise, increased calcium demand for muscle contraction and energy metabolism may stimulate PTH secretion, which, through *PTH1R*, enhances renal calcium reabsorption and skeletal calcium release to sustain calcium homeostasis (Goltzman and Hendy, 2015). *FGF23*, predominantly secreted by osteocytes and osteoblasts, regulates phosphate balance by modulating renal phosphate clearance and vitamin D metabolism (Lee et al., 2014; Liu et al., 2015; Hondares et al., 2014). By binding to the Klotho protein and *FGFR* receptor, *FGF23* initiates downstream signaling in target tissues including the kidneys, parathyroid glands, and bones (Wang et al., 2024; Shacham et al., 2023; Javier et al., 2025). According to Li et al. (Li et al., 2016), exercise-induced *FGF23* modulated skeletal muscle oxidative stress by limiting ROS overproduction and enhancing mitochondrial function to improve exercise performance. In the present analysis, significant upregulation of *FGF23* in post-race blood samples implicates its potential role in optimizing exercise performance through metabolic regulation in horses.

GO enrichment analysis revealed significant enrichment of differentially expressed mRNAs in BP and MF categories in the blood of Yili horses before and after racing. KEGG pathway analysis indicated marked enrichment in cAMP, MAPK, and PI3K-Akt signaling pathways. The cAMP pathway mediates intracellular signal transduction by modulating cAMP synthesis and degradation in response to extracellular stimuli, thereby influencing metabolic activity, gene expression, cellular differentiation, and survival (Glebov-McCloud et al., 2024; Zhang et al., 2024). Intense physical exertion may trigger an adrenaline surge that activates the cAMP-PKA axis, triggering glycogen phosphorylation, thereby enhancing muscle glycogen breakdown rate. Upon ligand binding to G protein-coupled receptors (GPCRs), adenylate cyclase (AC) is activated, catalyzing the conversion of ATP into cAMP and promoting energy production (Akizuki et al., 2021; Heckman et al., 2018). The MAPK pathway contributes to cellular proliferation and differentiation and is potentially associated with inflammatory signaling (Jia et al., 2025). The Mitogen-activated Protein Kinase (MAPK) pathway modulates cytokine generation (such as Interleukin-6) in cells during intense exercise in horses, mitigating muscle damage. The Phosphatidylinositol-3-Kinase/AKT cascade regulates cellular survival, growth, and metabolic function (Goyal et al., 2022). In the Iliyun horse race, IGF1 elevation is facilitated by stimulating proteoglycan synthesis to accelerate muscle repair, and by enhancing GLUT4 translocation to promote glycogen replenishment for metabolic adaptation. These pathways likely support energy homeostasis during intensive exercise and modulate post-race inflammatory responses in horses.

The PPI network constructed from DEGs in Yili horse blood pre- and post-race revealed that the top 10 core genes were all associated with heat shock proteins, exhibiting marked upregulation. *HSP90AA1* contributes to cellular stress responses and the maintenance of homeostasis (Goyal et al., 2022), while *HSPA4,* regulated by heat shock factor, is upregulated under stressors such as elevated temperature to maintain intracellular homeostasis (Li et al., 2019). Additionally, *KDM6B* is implicated in the modulation of inflammatory processes (Yang et al., 2024). These core genes may serve as candidate regulators influencing Yili horse race performance, although the underlying molecular mechanisms remain to be elucidated.

A total of 4,375 differentially expressed LncRNAs were detected in the peripheral blood of Yili horses pre- and post-race. The high expression levels observed across both time points suggested that these LncRNAs were not merely transcriptional byproducts of mRNA but may exert specific regulatory roles. GO enrichment of the predicted target genes revealed significant associations with biological regulation and metabolic processes. KEGG pathway analysis indicated enrichment in pathways such as Neuroactive ligand-receptor interaction, cAMP signaling, Calcium signaling, regulation of stem cell pluripotency, and cGMP-PKG signaling. Neuroactive ligand-receptor interactions have been implicated in mediating rapid cellular responses via ionotropic receptors (e.g., glutamate receptors) and GPCRs (Neele et al., 2018), In the 5000-meter race, LncRNAs might enhance neuronal excitability, boost muscle contraction precision and reaction speed, thereby supporting high-intensity endurance performance. In the present analysis, 124 differential LncRNAs were enriched in this pathway, suggesting their potential role in modulating neuromuscular responsiveness of Yili horses during racing. Additionally, intracellular Ca^2+^ serves as a critical signaling molecule regulating muscle contraction and neurotransmitter secretion within the calcium signaling pathway (Chan et al., 2025). LncRNAs may selectively target genes such as *RyR1*, modulating the release of calcium ions from the sarcoplasmic reticulum, thereby balancing explosive power with endurance. The enrichment of 88 differential LncRNAs in this pathway implies their involvement in exercise-related physiological processes, potentially by modulating neurotransmitter and endocrine hormone release during race conditions.

To investigate the association between circRNA expression and exercise in Yili horses, GO and KEGG enrichment analyses were conducted on the corresponding source genes. GO annotation revealed that the differentially expressed circRNA source genes were primarily linked to BP, with six genes implicated in nucleic acid circulation pathways. These results imply a potential involvement of circRNAs in modulating immune responses during exercise in Yili horses. KEGG analysis indicated that the source genes were predominantly enriched in metabolic and signal transduction pathways, including Axon guidance and T cell receptor signaling. Axon guidance relies on a combinatorial interaction of receptors and ligands to mediate cellular adhesion, attraction, or repulsion responses (de Almeida et al., 2016), During the competition, DEcirRNAs target genes such as *ROBO1/SLIT2* regulate axons of motor neurons, which may potentially affect muscle contractions. While T cell antigen receptors regulate the activation and proliferation of T lymphocytes (Seiradake et al., 2016). In this study, CircRNAs by modulating genes such as *CD3E*, inhibit excessive inflammatory responses post-exercise and promote tissue repair, thereby elucidating the molecular basis for the quick recovery seen in the Iliki horse after intense exercise, consistent with prior research (Masko et al., 2021), suggesting a functional contribution of the corresponding circRNAs to neural signaling and immune regulatory mechanisms.

Reverse transcription quantitative polymerase chain reaction (RT-qPCR) successfully confirmed seven mRNA trends; however, tissue-specific mechanisms such as heart muscle *HCN4* were not directly validated. In this study, we did not verify the identified DELncRNAs and DEcirRNAs by qRT-PCR, primarily due to the consistency between the pathways selected in our KEGG and GO enrichment analyses and those of the mRNAs. This led us to overlook the need for qRT-PCR analysis on LncRNAs and cirRNAs. We must admit that this indeed does affect our research on DELncRNAs and DEcirRNAs; in subsequent studies, we will need to delve deeper into these aspects. Despite the small sample size (n = 3), this study’s statistical analysis ensured reliability through stringent thresholds. In the data results displayed, the gene expression patterns remained clear, demonstrating the feasibility of the findings, and upon RT-qPCR validation, the reliability of the sequencing data was confirmed. This study has successfully established reliable data results in a small sample size, proving the feasibility and stability of the research technique.

## 5 Conclusion

In this study, transcriptome sequencing was employed to profile the whole transcriptome of Yili horse blood collected before and after a 5000-meter race. DEGs were predominantly enriched in the cAMP, MAPK, and PI3K-Akt signaling pathways, suggesting potential impact of racing on these items and signaling pathways. *HCN4, IGF-1, PTHR1*, and *FGF23* were annotated as exercise-related candidate genes in Yili horses. The findings offer a foundation for further exploration of candidate genes associated with equine exercise performance.

## Data Availability

The datasets presented in this study can be found in online repositories. The names of the repository/repositories and accession number(s) can be found below: https://www.ncbi.nlm.nih.gov/,PRJNA1250149.
